# Antibacterial and Phytochemical Screening of Artemisia Species

**DOI:** 10.3390/antiox12030596

**Published:** 2023-02-27

**Authors:** Maria-Evelina Bordean, Rodica Ana Ungur, Dan Alexandru Toc, Ileana Monica Borda, Georgiana Smaranda Marțiș, Carmen Rodica Pop, Miuța Filip, Mihaela Vlassa, Bogdana Adriana Nasui, Anamaria Pop, Delia Cinteză, Florina Ligia Popa, Sabina Marian, Lidia Gizella Szanto, Sevastița Muste

**Affiliations:** 1 Faculty of Food Science and Technology, University of Agricultural Sciences and Veterinary Medicine, 64 Calea Floresti, 400509 Cluj-Napoca, Romania; 2 Centre for Technology Transfer-BioTech, 64 Calea Florești, 400509 Cluj-Napoca, Romania; 3 Department of Medical Specialties, Faculty of Medicine, “Iuliu Hațieganu” University of Medicine and Pharmacy, 8 Victor Babeș Street, 400012 Cluj-Napoca, Romania; 4 Department of Microbiology, “Iuliu Hațieganu” University of Medicine and Pharmacy, 400012 Cluj-Napoca, Romania; 5 Raluca Ripan Institute for Research in Chemistry, Babeș-Bolyai University, 30 Fântânele Street, 400294 Cluj-Napoca, Romania; 6 Department of Community Health, “Iuliu Hațieganu” University of Medicine and Pharmacy, 6 Louis Pasteur Street, 400349 Cluj-Napoca, Romania; 7 9th Department-Physical Medicine and Rehabilitation, Carol Davila Univerity of Medicine and Pharmacy, 050474 București, Romania; 8 Physical Medicine and Rehabilitation Department, Faculty of Medicine, “Lucian Blaga” University of Sibiu, Victoriei Blvd., 550024 Sibiu, Romania; 9 Academic Emergency Hospital of Sibiu, Coposu Blvd., 550245 Sibiu, Romania; 10 Faculty of Biology and Geology, Babeș-Bolyai University, 44 Republicii Street, 400015 Cluj-Napoca, Romania

**Keywords:** *Artemisia absinthium* L., *Artemisia annua* L., antioxidants, sinapic acid, antibacterial

## Abstract

Taking into account the increasing number of antibiotic-resistant bacteria, actual research focused on plant extracts is vital. The aim of our study was to investigate leaf and stem ethanolic extracts of *Artemisia absinthium* L. and *Artemisia annua* L. in order to explore their antioxidant and antibacterial activities. Total phenolic content (TPC) was evaluated spectrophotometrically. Antioxidant activity was evaluated by DPPH and ABTS. The antibacterial activity of wormwood extracts was assessed by minimum inhibitory concentration (MIC) and minimum bactericidal concentration (MBC) in *Escherichia coli*, *Staphylococcus aureus*, *Listeria monocytogenes*, and *Salmonella enteritidis* cultures, and by zone of inhibition in *Klebsiella* carbapenem-resistant enterobacteriaceae (CRE) and *Escherichia coli* extended-spectrum β-lactamases cultures (ESBL). The *Artemisia annua* L. leaf extract (AnL) exhibited the highest TPC (518.09 mg/mL) and the highest expression of sinapic acid (285.69 ± 0.002 µg/mL). Nevertheless, the highest antioxidant capacity (1360.51 ± 0.04 µM Trolox/g DW by ABTS and 735.77 ± 0.02 µM Trolox/g DW by DPPH) was found in *Artemisia absinthium* L. leaf from the second year of vegetation (AbL2). AnL extract exhibited the lowest MIC and MBC for all tested bacteria and the maximal zone of inhibition for *Klebsiella* CRE and *Escherichia coli* ESBL. Our study revealed that AbL2 exhibited the best antioxidant potential, while AnL extract had the strongest antibacterial effect.

## 1. Introduction

There is an increasing interest in the antibacterial activity of herbal extracts, as they have been shown to be effective even on multidrug-resistant bacterial strains [1].

In 2015, the Nobel Prize for Physiology/Medicine was won by a doctor who discovered artemisinin (sesquiterpene lactone endoperoxide) to be an efficient treatment for malaria [2]. Artemisinin is a semisynthate found in all *Artemisia* plants in varying concentrations and is the most important active substance in *Artemisia annua* L. (*A. annua* L.) [3].

*A. annua* L., known as sweet wormwood, is a plant in the *Asteraceae* family that grows wild in Asia (primarily China, Japan, and Korea). It was imported to other countries such as Poland, Brazil, Spain, France, Italy, Romania, the United States, and Austria, where it was domesticated [4]. Since ancient times, Chinese herbalists have utilized it to treat a variety of ailments [5]. The mechanisms of action of *A. annua* L. and of artemisinin’s antimalarial effects is a current focus of research [6]. Furthermore, over the past few decades, research on *A. annua* L.’s effects on a variety of diseases, including inflammatory and cancerous conditions, and viral, bacterial, and parasite-related infections, has been performed [7,8].

*Artemisia absinthium* L. (*A. absinthium* L.), colloquially called wormwood, also belongs to the family *Asteraceae* (*Compositae*), of the tribe *Anthemideae*. It is a perennial, herbaceous, mesothermal, aromatic medicinal plant that has been used in traditional medicine from ancient times. The leaf and stem of wormwood, a very bitter-tasting plant, have traditionally been employed as a bitter tonic in appetite loss [9]. *A. absinthium* L.’s utility in gastrointestinal diseases is associated with its capacity to reduce the growth of microorganisms involved, due to its phenolic content [10].

There is an increasing interest in treating various degenerative diseases with different herbs and herbal extracts. Compounds with antioxidant activity, such as phenolic acids and flavonoids, are of interest to scientists as they can be further exploited [11]. Once elucidated, these compounds could be used in the pharmaceutical, cosmetic, and food industries [11,12].

The major bioactive antioxidant phenolic compounds found in *Artemisia* species are gallic acid, catechin, vanillic acid, caffeic acid, epicatechin, ferulic acid, sinapic acid, rutin, quercetin, luteolin, gentisic acid, chlorogenic acid, isoquercitrin, quercetol, kaempferol, and apigenin [13,14,15]. Phenolic compounds are widely distributed in plants, and they are associated with the prevention of several diseases in which oxidative stress plays an important role [16,17]. The amount of phenolic and flavonoid compounds in wormwood is positively correlated with its antioxidant capacity [18,19]. The health benefits of *Artemisia* include its antioxidant [20,21,22,23,24], neuroprotective [25], hepatoprotective [26], anti-inflammatory [27], renoprotective [28], and gastroprotective effects, and its digestive [29] and antibacterial activities [30,31,32]. The antibacterial activity of plants is a topic of interest for researchers because of the increasing antibacterial resistance to medications designed to kill them.

The objective of the current study was to investigate the phytochemical and antibacterial activities of leaf and stem ethanolic extracts of *A. absintium* L. and *A. annua* L. in different growing years.

## 2. Materials and Methods

### 2.1. Herb Samples and Ethanolic Extraction

The plant materials used in this study were the aerial parts (the stem and leaf) of *A. absinthium* L. and *A. annua* L. plants, from wild flora, collected at the end of June 2021, during the flowering period, from the outskirts of Blaj, Alba County, Romania. The samples were taxonomically authenticated at the University of Agricultural Sciences and Veterinary Medicine Cluj-Napoca, Romania. The plants were dried naturally at room temperature in a dark room without light (11 days). After drying, they were ground to powder. Ethanolic extracts from wormwood leaf and stem were obtained according to the method of Marțiș et al., 2021 [33], with some modifications. The dried material (1 g) was mixed with 50 mL of 96% ethanol for 24 h at 3–6 °C. Both samples were filtered through Whatman filter paper No. 4 and concentrated under reduced pressure at 35 °C, using a rotary evaporator (Rotavap Laborata 4010 Digital, Heidolph, Schwabach, Germany). The dried extract was recovered with 10 mL ethanol and stored at −18 °C until use. Three different replicates were performed for each extract’s extraction and all experiments were run in duplicates.

### 2.2. Reagents

Merck (Darmstadt, Germany) provided ethanol (HPLC grade), and Alpha Aesar, ThermoFisher provided 2,2′-Diphenyl-1-picrylhydrazyl radical (DPPH•, 95%) and 2,2′-Azino-bis (3-ethylbenzothiazoline-6-sulfonic acid) diammonium salt (ABTS, 98%). (ThermoFisher Kandel GmbH, Kandel, Germany). TCI supplied 6-hydroxy-2,5,7,8-tetramethylchroman-2-carboxylic acid (Trolox, 98%). (Portland, OR, USA). A Milli-Q Ultrapure water purification system was used to obtain analytical grade water (Millipore, Bedford, MA, USA).

### 2.3. Total Phenolic Content (TPC)

TPC was determined spectrophotometrically using the UV-Vis Specord 205 spectrophotometer (Analytik Jena GMbH, Jena, Germany) and Folin-Ciocalteu (FC) reagent, as previously published with minor changes [34]. In a 10 mL calibration flask, 0.4 mL of ethanol plant extract and 2 mL of FC reagent (diluted 1:1) were added. After shaking the mixture for 3 min, 1.6 mL of sodium carbonate solution (7.5%) was added. Water was used to bring the solution to volume. The solutions were cooled after having been exposed at 50 °C for 10 min, and the absorbance at 760 nm was measured against a reagent blank (0.4 mL water + 2 mL FC reagent + 1.6 mL sodium carbonate solution). TPC was estimated using the basis of the gallic acid calibration curve and reported as gallic acid equivalents (GAE) per gram of sample. TPC was also calculated in mg GAE/mL.

### 2.4. Assessment of the Antioxidant Activity

Assay for radical scavenging with DPPH. The antioxidant activity of plant extracts was assessed using the modified DPPH technique [35]. The extracts’ free radical scavenging activity was assessed in comparison to the effects of standard solutions of ethanol Trolox (6-hydroxy-2,5,7,8-tetramethylchroman-2-carboxylic acid) (0.02–0.1 mol/mL) or plant extracts on DPPH radical production, as follows: To 2 mL of ethanol and 0.5 mL of DPPH solution, 0.5 mL of each Trolox solution (or extract) was added. Then, 2.5 mL of ethanol was mixed with 2 mL of DPPH solution to make a control sample. The reactant mixture was vortexed for 30 s before being left to react in the dark for 30 min at room temperature. Each sample’s absorbance was recorded at 517 nm against a blank of ethanol. The antioxidant activity was calculated using the Trolox calibration curve and represented in micromoles per gram of material. Gallic acid was used as positive control.

Assay for radical scavenging ABTS•+. The antioxidant activity of plant extracts was determined using ABTS•+ in accordance with a previously established method [36] with adjustments. The procedure is based on the percentage inhibition of this radical’s peroxidation, which is visible as a darkening of a blue-green solution in an alkaline media at a wavelength of 734 nm. Amounts of 7 mM ABTS•+ solution and 2.45 mM potassium persulfate solution were included in the stock solutions. The working solution was then prepared by mixing equal parts of the two stock solutions and allowing them to react for 17 h at room temperature in the dark. After that, the solution was diluted by combining 1 mL of ABTS•+ solution with ethanol to obtain an absorbance between 0.700 and 0.800.

### 2.5. Quantitative Determination of Phenolic Compounds

The experiments of HPLC method [37] were conducted on a Jasco Chromatograph (Jasco International Co., Ltd., Tokyo, Japan) outfitted with a smart HPLC pump, an intelligent column thermostat, an intelligent UV/VIS detector, a ternary gradient unit, and an injection valve with a 20 µL sample loop (Rheodyne, Thermo Fischer Scientific, Waltham, MA, USA). The ChromPass^®^ software was used to process the experimental data (version v1.7, Jasco International Co., Ltd., Tokyo, Japan).

At 22 °C, a Lichrosorb^®^ RP-C18 column (25 × 0.46 cm) was used for separation, and UV detection was performed at 270 nm. The mobile phase was a solution of 0.1% formic acid and ethanol (A, HPLC grade). At a flow rate of 1 mL/min, the mobile phase was a mixture of ethanol (A, HPLC grade) and 0.1% formic acid solution (Millipore ultrapure water), and a gradient method was used: 0–10 min, linear gradient 10–25% A; 10–25 min, linear gradient 25–30% A; 25–50 min, linear gradient 35–50% A; 50–70 min, isocratic 50% A. The injection volume was constantly 20 µL. The compounds were identified by comparing their elution periods to the ones of the standard compounds examined under the same HPLC circumstances.

Standards solutions. In ethanol, a stock standard solution (1 mg/mL of each) was produced and kept at 4 °C. The calibration curves were generated with four concentration levels ranging from 120 g/mL to 11.25 g/mL, with R2 values greater than 0.998.

### 2.6. Antibacterial Activity

#### 2.6.1. Determination of the Minimum Inhibitory Concentration (MIC)

*Escherichia coli* ATCC 25922, *Staphylococcus aureus* ATCC 25923, *Salmonella enteritidis* ATCC 13076, and *Listeria monocytogenes* ATCC 19114 were examined as standard strains. They were cultivated for 24 h at 37 °C in a test tube containing 10 mL of sterile nutritional broth (Oxoid Ltd., Basingstoke, Hampshire, England). TBX agar was used for *E. coli*, BP agar for *S. aureus*, XLD agar for *S. enteritidis* (Oxoid Ltd., Basingstoke, Hampshire, England), and Palcam agar base enhanced with *Listeria* Palcam antimicrobic supplement (Oxoid Ltd., Basingstoke, Hampshire, England) for *L. monocytogenes*. Plates were incubated at 37 °C for 24 h. Optical microscopy was used to confirm bacterial morphology. Several colonies of each strain were distributed into sterile saline solution and corrected to match the turbidity of the McFarland 0.5 standard (1.5 × 10^8^ CFU/mL) grown on Mueller–Hinton agar (Oxoid Ltd., Basingstoke, Hampshire, England). Then, for each microplate well, a 1.5 × 10^6^ CFU/mL bacterial suspension was produced [38]. The MIC was obtained using a microtiter plate-based antibacterial test based on resazurin [39]. A 96-well microplate was filled with 100 µL of sterile nutritional broth (Oxoid Ltd., Basingstoke, Hampshire, England). The material was then added to the first well in 100 µL increments, and serial 11-fold dilutions were made in the remaining wells of each row by moving 100 µL from well to well. The excess 100 µL in the row’s final well was discarded. Then, in each well, 10 µL of inoculum (1.5 × 10^6^ CFU/mL) was introduced. The positive control was gentamicin (0.04 mg/mL in saline solution), while the negative control was ethanol 96%. The microplates were incubated for 20–22 h at 37 °C, then 20 µL of 0.2 mg/mL resazurin aqueous solution was added into each well. The microplates were subsequently incubated for 2 h at 37 °C. After this period, resazurin (a blue nonfluorescent dye) was oxidized to resorufin (fluorescent pink) wherever viable bacterial cells were present. As a result, the concentration in the last well remained blue in each row and was considered to totally block bacterial growth and represented the MIC. For each sample, two replicates were performed.

#### 2.6.2. Assessment of the Minimum Bactericidal Concentration (MBC)

The MBC was determined by plating a 10 µL aliquot on solid culture Mueller–Hinton medium from the last three wells that demonstrated inhibition of bacterial growth in the MIC assay (Oxoid Ltd., Basingstoke, Hampshire, England). The plates were incubated at 37 °C for 24 h. The MBC was defined as the lowest concentration that stopped bacterial growth (no colonies on the plate). For each plate, three separate biological replicates were performed, and all experiments were run in duplicate.

### 2.7. Bacterial Samples

Isolation of the bacterial strains was performed using selective chromogenic media CHROMID^®^ ESBL (bioMérieux, Marcy-l’Étoile, France) and CHROMID^®^ OXA-48 (bioMérieux, Marcy-l’Étoile, France). To identify the colonial morphology, the isolated strains were inoculated into the following media: Columbia blood agar (bioMérieux, Marcy-l’Étoile, France) and MacConkey agar (bioMérieux, Marcy-l’Étoile, France). The biochemical tests used for identification were as follows: TSI (triple sugar iron), MIU (motility indole urea), SIM (sulfide indole motility), citrate, PAD (phenylalanine deaminase), and OF (oxidation–fermentation). Only the strains that matched the colonial morphologies and biochemical profiles of *Klebsiella* spp. and *E. coli* were used later in this experiment. The antibiotic susceptibility profiles were determined using the Kirby–Bauer disk diffusion method. Moreover, the CRE (carbapenem-resistant enterobacteriaceae) phenotype was identified using the Diatabs™ (Rosco Diagnostica) disk diffusion synergy test on Mueller–Hinton agar. A total of 15 strains were used in this study: 5 strains of *Klebsiella* spp. ESBL, 5 strains of *Klebsiella* spp. CRE, and 5 strains of *E. coli* ESBL. Pathogens were obtained from the Department of Microbiology collection (Iuliu Hatieganu University of Medicine and Pharmacy Cluj-Napoca).

To test the antibacterial effects of the studied extracts, antibiograms were performed using the Kirby–Bauer disk diffusion method [1,40]. Mueller–Hinton media were inoculated with 0.5 McFarland bacterial suspensions using the spread plate technique. Sterilized filter paper disks, 5.49 mm in diameter, imbued with 5 microliters of the studied extracts, were used to evaluate the antibacterial activity. The media were incubated at 37 °C for 24 h, and the zones of inhibition were measured thereafter.

### 2.8. Statistical Analysis

The ANOVA analysis of variance was used to compare the average mean values, with a confidence interval of 95% or 99%, using SPSS 19.0 statistical analysis (IBM, New York, NY, USA) and Tukey’s honestly significant differences (HSD) test. A *p*-value of less than 0.05 was deemed statistically significant.

## 3. Results

### 3.1. Total Phenolic Content of Different Tissues of Artemisia Extracts

Table 1 is a summary of the phenolic content found in the different tissues of the *A. annua* L. and *A. absinthium* L. samples. It is striking that these components were higher in the leaf from the second year of vegetation than in all the other parts examined in this study. For the *A. annua* L. leaf (AnL), we measured 518.09 ± 0.01 mg GAE/mL.

### 3.2. Antioxidant Activity

Two different chemical methods, namely, DPPH and ABTS assays, were used to assess the antioxidant activity of the studied wormwood extracts (Table 1). The leaf and stem of *A. absinthium* L. extracts from the second year of vegetation had higher values as compared to the leaf extract from the first year. The values obtained using ABTS were higher than those obtained using DPPH. As expected, the levels of antioxidant capacity were significantly different (*p* < 0.001) among different plant tissues for both *Artemisia* species.

The intensity of relationships between the TPC, DPPH, and ABTS was determined with Pearson’s correlation with a 95% confidence interval. Statistically significant correlations were established (*p* ≤ 0.05) between the DPPH free radical scavenging activity and TPC values in the leaf of both *Artemisia* species (r = 0.967) using Pearson’s correlation test (Table 2).

### 3.3. Phenolic Compound Profile by HPLC

Phenolic compounds from plants such as rutin, catechin, and ferulic acid have received increasing interest due to their potential antioxidant activity. Eleven phenolic compounds were found in the *Artemisia* species (Table 3, Supplementary Materials using a chromatographic analysis of the ethanolic extracts). Two phenolic acids, i.e., vanillic acid and p-coumaric acid, were present in both the leaf and stem.

From the flavan-3-ols, epicatechin was the dominant compound in *A. annua* L. and *A. absinthium* L.

### 3.4. Antibacterial Activity of Different Tissues of Artemisia

The results of the antibacterial assay are presented in Table 4. The following standard strains were tested: *Escherichia coli* ATCC 25922, *Staphylococcus aureus* ATCC 25923, *Salmonella enteritidis* ATCC 13076, and *Listeria monocytogenes* ATCC 19114. The ethanolic extract of the *A. annua* L. leaf exhibited antibacterial activity against all the bacterial strains tested. The MIC of the ethanolic extract of AnL ranged from <2.00 ± 0.014 mg/mL (against *S. aureus* ATCC 25923) to 375.00 ± 0.014 mg/mL AbS1 (against *E. coli* ATCC 25922 and *S. enteritidis* ATCC 13076). The MBC of the *A. annua* L. ethanolic extract ranged from 5.00 ± 0.014 mg/mL (against *S. aureus* ATCC 25923 and *L. monocytogenes* ATCC 19114) to 375.00 ± 0.014 mg/mL AbS1 (against *S. aureus* ATCC 25923, *E. coli* ATCC 25922, *S. enteritidis* ATCC 13076, and *L. monocytogenes* ATCC 19114).

The antibacterial effects exhibited by wormwood were significant as compared to the ethanol 96% controls (*p* < 0.05) (Table 5). Our findings show that the ethanolic extracted from *A. annua* L. and *A. absinthium* L. possessed significant antibacterial effects against *Klebsiella* ESBL, *Klebsiella* CRE, and *E. coli* ESBL.

## 4. Discussion

### 4.1. Phytochemical Characteristics of Extracts

The antioxidative effects of plants in the *Artemisia* genus are most probably due to the high amounts of phenolic compounds.

The polyphenolic profiles of the extracts from the leaf and stem of *Artemisia* species were assessed as a source of natural antioxidants.

The highest values of TPC were found in AnL (518.09 ± 0.01 mg GAE/mL) and in AbL2 (487.36 ± 0.08 mg GAE/mL). Intermediate values were determined for AbL1 (229.68 ± 0.16 mg GAE/mL) and AnS (135.34 ± 0.08 mg GAE/mL). The lowest values were found in AbS2 (60.59 ± 0.20 mg GAE/mL) and in AbS1 (51.73 ± 0.11 20 mg GAE/mL). In our study, ethanolic extracts of leaves had higher TPC than ethanolic extracts of stems, regardless of the species and year of vegetation.

Lee proposed three other extraction methods for *A. absinthium* L. leaf from South Korea: ethyl acetate, methanol, and water [41]. The best TPC extraction rate was found for the aqueous leaf extract at 134.47 ± 0.016 mg/100 g DW. Since the values obtained in the present study are higher (Table 1), we believe ethanol extraction to be more efficient than the solvents listed above. Similar results were recently communicated by Sembirin, who showed that the amount of antioxidant compounds extracted from *A. annua* L using an ethanol solvent was higher than the amount of antioxidant compounds extracted by a methanol solvent [42]. A higher value was also observed for the hydroalcoholic extract of *A. absinthium* L. from India, obtained from the aboveground parts of the plant (9.29 ± 0.51 mg GAE/g DW), as compared to hexane (0.43 ± 0.07 mg GAE/g DW) and methanol (3.55 ± 0.39 mg GAE/g DW) extracts [43].

In previous studies, it was shown that the strength of antioxidant activity was influenced by the content of phenolic compounds and total flavonoids [8,34,35].

In our study, the antioxidant activity was not directly proportional to the TPC from the leaf and stem ethanolic extracts of each individual species of *Artemisia*. Only in the global analysis of the leaf extracts was a high positive correlation found between TPC and antioxidant activity for both types of determinations, DPPH and ABTS (Table 2). Among the analyzed extracts, AbL2 had the highest antioxidant capacity for both determination methods (ABTS and DPPH). We found that the decreasing order of antioxidant activity among the different extracts from the aerial parts of wormwood was the following: AbL2 (735.77 ± 0.02 µM Trolox/g DW) > AnL (250.51 ± 0.01 µM Trolox/g DW) for DPPH method; AbS1 (1314.38 ± 0.01) > AnS (659.57 ± 0.02 µM Trolox/g DW) for the ABTS method. Previously published studies also found that the antioxidant capacity was not necessarily directly proportional to the amount of active compounds from the plant. Minda showed that *Artemisiae annue herba* had an antioxidant activity (DPPH method) of 24.14 ± 0.6% at a 50 µg/mL concentration and reached only 90.04 ± 2.25% at a 1000 µg/mL concentration [15].

Concerning the growing year of the *A. absinthium* L. leaf, there was an approximate 12-fold increase in the antioxidant activity in the second year as compared to the first year (the DPPH method).

Comparing the two varieties of wormwood in the first year of growth, the antioxidant activity was significantly higher in *A. annua* L. as compared to *A. absinthium* L. for both methods used (DPPH and ABTS). However, for the stem in the first year of vegetation, the order of the varieties changed in favor of *A. absinthium* L. (AbS1 > AnS). Moreover, the antioxidant activity of the *A. absinthium* L. stem extract was slightly higher than that of the *A. absinthium* L. leaf extract in the first year of vegetation. These results are consistent with the study of Moacă [9].

Sengul obtained the highest total phenolic content in *A. absinthum* (9.79 µg GAE/mg), followed by *A. santonicum* (15.38 µg GAE/mg) and *Saponaria officinalis* (6.57 µg GAE/mg), with a positive correlation (r = 0.819) between the antioxidant activity and the TPC of the plant samples [44].

Many studies reported a strong relationship between the TPC and the antioxidant activity in certain plant products [19,21,33,44,45,46]. In our study, we found a positive and extremely high correlation (r = 0.959, *p* = 0.0001) between the TPC and the antioxidant activity (DPPH) when all plant extracts were taken into account. When leaf and stem extracts were individually assessed, the correlation coefficient between TPC and DPPH was r = 0.967 (*p* = 0.0001) for the wormwood leaf samples. Unexpectedly, an extremely high negative correlation (r = −0.949, *p* = 0.001) was found between the TPC and DPPH for the stem.

On the basis of the HPLC analysis and the calibration curves of the standard samples, the phenolic compound content was determined in all extracts (Table 3). The leaf extract of *A. annua* L. was found to be the richest in bioactive compounds (AnL > AnS), and it had high levels of sinapic acid (285.694 ± 0.002 µg/mL), p-coumaric acid (51.267 ± 0.002 µg/mL), vanillic acid (46.863 ± 0.002 µg/mL), and rutin (17.320 ± 0.000 µg/mL).

Sinapic acid (3,5-dimethoxy-4-hydroxycinnamic acid) is the most representative phytochemical hydroxycinnamic acid of the flavonoid compounds identified. In accordance, the results of Baiceanu also indicated high levels of sinapic acid in other herbal extracts of the *Artemisia* genus (*A. abrotanum* L. 79.95 µg/mL) [47]. Sinapic acid is a natural compound with various potential health benefits, including antibacterial [48], antioxidant [49,50], anti-inflammatory [51,52], antihypertensive, cardioprotective [53], anxiolytic [54], and antiaging effects [55].

Quercetin, which is a flavonol, was identified in only two extracts. The highest amount was found in AbS1 (8.492 ± 0.002 µg/mL), and the lowest in AnL (1.653 ± 0.003 µg/mL). Quercetin possesses antioxidant, anti-inflammatory, neuroprotective, cardioprotective, antiobesity, anticancer, and antimicrobial activity. Quercetin antimicrobial activity against Gram-positive and Gram-negative bacteria, including various drug-resistant microorganisms, could be explained by its capacity to damage microbial cell membrane, to inhibit nucleic acids and proteins synthesis, to reduce expression of virulence factors and to prevent biofilm formation [56].

Epicatechin possesses antioxidant, anti-inflammatory, vasoprotective, neuroprotective, anticancer, and antimicrobial activity [57]. In a previous study, epicatechin demonstrated an inhibitory effect on *Helicobacter pylori* growth [58]. In our study, the highest content of epicatechin was recorded in the AbS2.

Gallic acid is another hydroxybenzoic acid that modulates the immune response and antimicrobial natural defense. Due to its antimicrobial activity, gallic acid is used to synthesize trimethoprim, an antimicrobial agent [59]. In our study, gallic acid was found in a higher amount in AnL (1.132 ± 0.001 µg/mL) and in a lower amount in AnS (0.086 ± 0.004 µg/mL), but it was not detected in the *A. absinthium* L. extracts. In contrast, in a study focused on the chemical compounds in the aerial part of Romanian *A. absinthium* extracts, a small amount of gallic acid (0.092 ± 0.005 mg/g DW) was observed [46]. In an *A. absinthium* leaf extract from South Korea, Lee found the amount of gallic acid to be 63.99 ± 0.827 µg/g [41].

Vanillic acid is an antioxidant monohydroxybenzoic acid with antimicrobial activity against *S. aureus* and *E. coli* [60]. The highest amount of vanillic acid was identified in AbL2 (66.777 ± 0.002 µg/mL) and the lowest was recorded in AbS1 (2.420 ± 0.002 µg/mL). The decreasing order of samples for vanillic acid was as follows: AbL2 >AnL > AbL1 > AbS2 > AnS > AbS1.

Caffeic acid was not detected in any sample from the stem or leaf from either variety. The antioxidant activity of certain phenolic acids indicated the following order: caffeic acid > sinapic acid > ferulic acid > p-coumaric acid.

p-Coumaric acid has antioxidant and bactericidal activity based on DNA and bacterial cell membrane damages [61]. In the present study, the highest amount of p-coumaric acid was found in the AnL (51.267 ± 0.002 µg/mL). Another p-coumaric acid value reported for wormwood leaf extracts was 9.10 ± 0.141 µg/g [41].

Ferulic acid acts as a superoxide scavenger, similarly to superoxide dismutase [62]. It also exhibited anti-inflammatory, antidiabetic, and anticancer effects and antimicrobial activity [63]. In our study, the ferulic acid amount in the analyzed samples was low, in accordance with the findings of other studies [41,46].

The differences between the phenolic compound values in the leaf and stem of *A. absinthium* L. were evident for the wormwood extracts in the first year of growth. The amounts of phenolic compounds were higher in AbL1 (42.241 ± 0.001 µg/mL for vanillic acid, 13.488 ± 0.001 µg/mL for epicatechin, and 2.565 ± 0.002 µg/mL for p-coumaric acid) than in AbS1. The exception was catechin, whose value was higher in the stem extract than in the leaf extract (2.438 ± 0.002 µg/mL in AbS1, 1.262 ± 0.002 µg/mL in AbL1), indicating a higher antioxidant activity in the stem.

Differences between the phenolic profile of the leaf and the stem ethanolic extracts of *A. absinthium* L. were also observed in the second year of growth. The amounts of phenolic compounds were higher in AbL2 (66.777 ± 0.002 µg/mL for vanillic acid and 1.375 ± 0.002 µg/mL for p-coumaric acid) than in AbS2. Epicatechin was found in high amounts in AbS2 (21.123 ± 0.001 µg/mL).

Flavonoids, such as rutin, quercetin, epicatechin, and catechin, involved in free-radical scavenging activity were also reported in other studies focused on wormwood extracts. Some additional phytocompounds were identified in other studies, including gentisic acid, chlorogenic acid, caffeic acid, isoquercitrin, quercetol, kaempferol, and apigenin [15]. Free-radical scavenging activity and anti-inflammatory activity was demonstrated for these pharmacophores [64,65,66,67,68].

### 4.2. Antibacterial Effects

The inhibitory activity of herbal extracts against Gram-positive bacteria, especially *S. aureus*, has been widely reported in the literature [69]. Wormwood’s significant antibacterial activity against surgical wounds infected with *S. aureus* (the most common cause of surgical wound infections) in a rat model was reported by Moslemi [31]. In our study, the highest antibacterial activity (MIC = 2.00 ± 0.014 mg/mL and MBC = 5.00 ± 0.014 mg/mL) against *S. aureus* was recorded for AnL extract. The AnL antibacterial activity could be attributed to the sinapic acid (285.694 ± 0.002 mg/mL). Previous in vivo studies conducted on Gram-positive and Gram-negative bacteria found that sinapic acid exhibited significant antibacterial activity against various microorganisms [55].

AbL2 extract was found to be in the second place with its efficacy against *S. aureus*, with MIC = 25.00 ± 0.002 mg/mL and MBC = 54.00 ± 0.014 mg/mL. For AnS, with MIC = 54.00 ± 0.002 mg/mL and MBC = 114.00 ± 0.014 mg/mL, the antibacterial activity was also shown to be satisfactory.

The antibacterial activity against S. *aureus* for AbL2, in which the phenolic compounds gallic acid, caffeic acid, ferulic acid, sinapic acid, rutin, quercetin, and luteolin were not detected, could be due to vanillic acid. In a previous study, Keman showed the importance of vanillic acid for treatment in methicillin-resistant *S. aureus* infections [70].

The antimicrobial effects against *S. aureus* found for AbS2 (MIC = 114.00 ± 0.014 mg/mL, MBC = 114.00 ± 0.014 mg/mL), AbL1 (MIC = 89.50 ± 0.028 mg/mL, MBC = 255.00 ± 0.014 mg/mL), and AbS1 (MIC = 85.00 ± 0.002 mg/mL, MBC = 375.00 ± 0.014 mg/mL) were low.

The chemical composition of *A. absinthium* L. differs according to geographical area [71], the physiological part of the plant [72], the temperature [73], and the degree of senescence [74]. For this reason, there is no standard chemical composition and antibacterial activity.

Valdes studied the antibacterial activity of Cuban medicinal plants, wormwood ethanolic extract included, and found no antibacterial activity related to the wormwood extract for the concentrations tested (64 µg/mL to 0.25 µg/mL) [75]. In another study, Fiamegos also demonstrated that chloroform extracts from *A. absinthium* L. leaf (in a concentration range of 150–500 mg/mL), tested on pathogenic bacteria, had no antibacterial activity against *E. coli* but inhibited *S. aureus* [30].

Another pathogenic bacterium used to test the efficacy of the wormwood extracts in our study was *E. coli*. We noticed similarities between the activity against the two pathogenic bacteria: *S. aureus* and *E. coli*. The most pronounced antibacterial activity against *E. coli* was identified for the AnL extract, with MIC = 5.00 ± 0.014 mg/mL and MBC = 12.00 ± 0.014 mg/mL. It has to be noted that this extract contains a wide range of phenolic compounds. Thus, the extract’s antibacterial effect could be correlated to them. We also found significant antibacterial activity in AbL2 (MIC = 54.00 ± 0.014 mg/mL and MBC = 54.00 ± 0.002 mg/mL) and in AnS (MIC = 54.00 ± 0.014 mg/mL and MBC = 114.00 ± 0.014 mg/mL). A low *E. coli* inhibition activity was recorded for AbL1 (MIC = 255.00 ± 0.014 mg/mL and MBC = 255.00 ± 0.014 mg/mL), similarly with AbS2 (MIC = 240.00 ± 0.014 mg/mL and MBC = 240.00 ± 0.014 mg/mL). The extract with the weakest antibacterial activity was that from AbS1 (MIC = 375.00 ± 0.014 mg/mL, MBC = 375.00 ± 0.014 mg/mL).

A lot of other researchers identified the antibacterial activity of different wormwood species. In his research, Baykan Erel demonstrated the moderate effect of *A. absinthium* L. methanolic extract on *E. coli* ATCC29998 and on *E. coli* ATCC11230, with 10 mm and 7 mm inhibition zones, respectively [76]. Sengul also reported antibacterial activity for two types of extracts: aqueous and methanolic, from the aerial parts of *A. absinthium* L. The inhibition zones reported for *S. aureus* ATCC29213 were 12 mm for the aqueous extract and 15 mm for the methanolic extract; meanwhile, for *E coli* 1328, the inhibition zones were weaker: 7 mm for the aqueous extract and 11 mm for the methanolic extract [44].

Mihajilov-Krstev demonstrated that the MIC of *A. absinthium* L. essential oil ranged from <0.08 mg/mL for *S. aureus* isolated from the wound to 0.30 mg/mL against *S. aureus* ATTC 25923. The MBC of *A. absinthium* L. essential oil ranged from <0.08 mg/mL for *S. aureus* isolated from the wound to 0.61 mg/mL against *S. aureus* ATTC 25923. The same author showed that the MIC of *A. absinthium* L. essential oil ranged from 1.21 mg/mL for *E. coli* isolated from stool and against *E. coli.* (ATTC) 8739 to 0.39 mg/mL for *E. coli* isolated from wounds. The MBC of *A. absinthium* L. essential oil ranged from 2.43 mg/mL for *E. coli* isolated from stool to 2.43 mg/mL for *E. coli* isolated from wounds and against *E. coli* ATTC 8739. In the same study, the MIC of *A. absinthium* L. essential oil against *L. monocytogenes* was 0.30 mg/mL and the MBC was 38.80 mg/mL [77]. Sultan demonstrated the antibacterial activity against *E. coli* and *S. aureus* bacteria for a reaction mixture prepared by dissolving hot methanolic extract *A. absinthium* L. leaf in Milli-Q water [78]. Lopes-Lutz demonstrated that *A. absinthium* L. essential oil was one of the most active agents against *Staphilococcus* strains and that it also had antibacterial activity against *E coli*. The zone of inhibition expressed in millimeters for *S. aureus* was high (25 ± 1.4 mm), confirming our results, while it was significantly lower (5 ± 0.0 mm) for *E. coli* [79]. Msaada studied the antibacterial activity of *A. absinthium* L. essential oil from four different areas of Tunisia. The highest antibacterial activity was recorded against *S. aureus* 25923 for the essential oil from Kairouan (25 ± 1.13 mm diameter of inhibition), followed by those from Jerissa (diameter of inhibition of 20.66 ± 0.65 mm), Boukornine (diameter of inhibition 20.66 ± 2.61 mm) and Bou Salem (18 ± 1.13 mm diameter of inhibition) [71]. On the other hand, Joshi, who also studied the activity of *A. absinthium* L. essential oil from India, did not identify any antibacterial activity for *S. aureus* and *E. coli* [80]. Likewise, Jouteau, tested the antibacterial activity of *A. absinthium* L. essential oil against *S. aureus* (CIP 53154) and *E. coli* (CIP54127) using the liquid diffusion method and found no effect at the tested concentrations [81].

The antibacterial activity of the extracts against *S. enteritidis* ATCC 13076 was also investigated. As compared to the other bacteria assessed in this study, poor antibacterial activity against this bacterium was evident. We observed that the AnL extract exhibited a good action against all bacteria, and *S. enteritidis* was no exception, with MIC = 5.00 ± 0.014 mg/mL and MBC = 12.00 ± 0.014 mg/mL. Moreover, AbS1 had the weakest antibacterial activity against all bacteria considered, with MIC = 375.00 ± 0.014 mg/mL and MBC not detected for *S. enteritidis* ATCC 13076. A moderate antibacterial activity was identified for AbL2, with MIC = 54.00 ± 0.002 mg/mL and MBC = 54.00 ± 0.002 mg/mL. Low antibacterial activity was also identified for AbL1, with MIC = 255.00 ± 0.014 mg/mL and MBC not detected. On the other hand, Kordiali found no antibacterial activity against *S. enteriditis* ATCC 13076, *S. aureus* ATCC 29213, or *E. coli* for the essential oil obtained from aerial parts of *A. absinthium* L. from Turkey [18]. Based on our research and the aforementioned studies, we can state that the plant origin area and the type of extract used (ethanolic, methanolic, aqueous, etc.) can cause differences in the antimicrobial activity of wormwood extracts.

In order to support the above mentioned findings, Msaada studied the antibacterial activity of *A. absinthium* L. essential oil from four different areas of Tunisia against *L. monocytogenes* ATCC 19195 and found different inhibitory areas: 20.00 ± 1.13 mm and 20.00 ± 1.95 mm were recorded for the essential oil from the Bou Salem and Kairouan areas (the highest values), 18.66 ± 2.35 mm for the essential oil from Boukornine, and 17.33 ± 1.72 mm for the essential oil from the Jerissa area [71].

In our study, the best antibacterial activity against *L. monocytogenes* ATCC 19114 was found in AnL, with MIC = 5.00 ± 0.014 mg/mL and MBC = 5.00 ± 0.014 mg/mL. The MIC against *L. monocytogenes* ATCC 19114 of the ethanolic extract of *A. absinthium* ranged from 54.00 ± 0.002 mg/mL (AbL2) to 178.00 ± 0.014 mg/mL (AbS1). The year of vegetation positively influenced the MIC and MBC values of *A. absinthium* L. The leaf and stem of *A. absinthium* L. from the second year of vegetation had better mean MIC and MBC values than those from the first year (e.g., MIC = 54.00 ± 0.002 mg/mL for AbL2 and MIC = 121.00 ± 0.014 mg/mL for AbL1). As compared to MIC (0.3 mg/mL) and MBC (38.80 mg/mL) against *L. monocytogenes* ATCC 7644 of the essential oil from the *A. absinthium* family harvested from Serbia, the value was higher in our sample [77].

*L. monocytogenes* ATCC 19114 was differently influenced by the ethanolic stem extracts of the plants included in this study. Thus, the *A. annua* L. and *A. absinthium* L. stem samples from the second year of vegetation exhibited better antibacterial activity than those from the first year (AnS = AbS2 > AbS1). This could be explained by the fact that there was approximately twice the amount of polyphenols and flavonoids in the leaf vs. stem (Table 5), which is in accordance with other studies [82].

In an *A. annua* L. leaf studied until senescence, Lommen found the maximum amount of artemisinin in the leaf after the onset of senescence [74]. In our study, the amount of vanillic acid, ranging from 66.777 ± 0.002 mg/mL in the second year to 42.241 ± 0.001 mg/mL in the first year, was responsible for the antibacterial activity, which was significantly higher for the second year of vegetation in the AbL2 extract on all strains tested (*S. aureus* ATCC 25923, *E. coli* ATCC 25922, *L. monocytogenes* ATCC 19114, and *S. enteritidis* ATCC 13076).

Antibacterial activity against carbapenem-resistant enterobacter hormaechei (CREH) as mediated by vanillic acid was studied by assessing variations in the intracellular ATP concentration, intracellular pH, and membrane potential [83]. Moreover, the addition of vanillic acid (500 µg/mL vanillic acid 65%) to the growth medium of *S. marcescens* ATCC 14756 and MG1 significantly affected biofilm production and virulence in a concentration-dependent manner [84]. Vanillin, ethyl vanillin, and vanillic acid may be useful for controlling *Cronobacter* spp. in food during preparation and storage, and disrupting the cell membrane of CREH [85]. Various *Artemisia* species were shown to produce metabolites with antibacterial activity. Furthermore, in the ethanolic extract, a high level of chlorogenic acid was found in a tall species of the genus *Asteraceae* (*A. gmelinii*). Recent studies show that chlorogenic acid bonds to the outer membrane, disrupts it, depletes the intracellular potential, and releases macromolecules from the cytoplasm, leading to cell death [14].

*Artemisia* extracts exhibited potent antibacterial activity against selected clinically-important pathogenic bacteria as judged by the low MIC values. The results of the present study demonstrate the significant antibacterial activity of wormwood ethanolic extract against *Klebsiella* spp. ESBL, *Klebsiella* spp. CRE, and *E. coli* ESBL (Table 5).

Our findings indicate that AnL, AbL1, and AbS1 from the first year of vegetation had significant activity against *Klebsiella* ESBL (10.863 ± 0.308 mm for AnL; 10.110 ± 1.68 mm for AbL1; 11.246 ± 1.71 mm for AbS1). The vanillic acid and epichatechin found in the aforementioned samples conferred antibacterial properties to the extracts. It is well-established that flavonoids have multiple hydroxyl groups and, therefore, have a pronounced potential to bind proteins. The inhibition of the binding affinity of KpDnaB to dNTPs (deoxyribonucleoside triphosphate) in Klebsiella pneumonia by flavonols could explain their antibacterial activity [86].

Significant antibacterial action against *Klebsiella* CRE was found in samples of *A. annua* L., both in the leaf and stem, with sinapic acid well-represented in the extracts (12.756 ± 0.993 mm for AnL; 9.843 ± 0.945 mm for AnS). AnL and AnS extracts appeared to have the best effect against *E. coli* ESBL (8.610 ± 1.861 mm, 5.67 ± 0.682 mm, respectively). The antibacterial activity of sinapic acid was demonstrated in various studies on both plant and human pathogens [87], including *E. coli* [88]. As a result of their capacity to form hydrogen bonds with amino-acid residues of theactive site of the NorA efflux pump, sinapic acid exhibits a significant antibacterial activity against the NorA-bearing Gram-positive and Gram-negative bacteria, S aureus, and E. coli. Moreover, they can be safely administered orally and can penetrate the cell wall to reach the NorA active site [89].

### 4.3. Limits of the Study

In addition to the methods used by us (DPPH for stable radicals and ABTS for cation radicals), other methods for determining the antioxidant activity of wormwood extract should also be used, such as chemical-based methods (the cupric ions reducing power assay and ferric reducing antioxidant power) or biological assays (cellular antioxidant activity assay) [90].

In order to draw firm conclusions concerning the influence of the vegetation year, both *Artemisia* species should be analyzed in the first and in the second year.

## 5. Conclusions

The year of vegetation, the part of the plant, and the species influenced the TPC of the wormwood extract. The highest value was obtained for the leaf sample in the second year of vegetation. As regards the species, *A. absinthium* L. registered the highest TPC, with leaf superior to stem. Sinapic acid was abundant in *A. annua* leaf/stem extracts. In all ethanol leaf samples, vanillic acid was present in significant amounts. Concerning the activity of wormwood extracts against *S. aureus,* the results showed that the leaf, rich in phenolic compounds, had a higher antibacterial activity than the stem.

The antibacterial activity against *S. aureus* depended on the growing year of the plants. *A. absinthium* extracts from the first year of vegetation exhibited a weaker antibacterial activity than *A. absinthium* extracts from the second year. The *A. annua* L. species, rich in polyphenolic compounds, mainly in the leaf, was proven to have antibacterial activity against *Salmonella enteritidis*. From all the *Artemisia* extracts studied, AnL and AnS exhibited significant activity against *Klebsiella* spp. CRE and *E. coli* spp. ESBL. Thus, on the basis of our results and the recent literature, the application of new therapeutic protocols for resistant infectious diseases based on the use of natural extracts of *Artemisia* is a real possibility and should be further studied.

## Figures and Tables

**Table 1:** Antioxidant activity of wormwood ethanolic aerial parts, depending on the year of vegetation.

Botanical Family	Herb	Year of Vegetation	Sample	TPC [mg GAE/mL Extract]	TPC [mg GAE/100 g DW]	DPPH [µM Trolox/g DW]	ABTS [µM Trolox/g DW]
*Asteraceae*	*Artemisia annua* L. leaf	I	AnL	518.09 ± 0.01 ^a^	2089.07 ± 0.03 ^b^	250.51 ± 0.01 ^b^	816.55 ± 0.05 ^d^
	*Artemisia annua* L. stem	I	AnS	135.34 ± 0.08 ^d^	390.77 ± 0.03 ^c^	60.87 ± 0.02 ^e^	659.57 ± 0.02 ^e^
	*Artemisia absinthium* L. leaf	II	AbL2	487.36 ± 0.08 ^b^	3778.512 ± 0.02 ^a^	735.77 ± 0.02 ^a^	1360.51 ± 0.04 ^a^
	*Artemisia absinthium* L. stem	II	AbS2	60.59 ± 0.20 ^e^	299.21 ± 0.02 ^e^	129.49 ± 0.01 ^c^	1118.12 ± 0.02 ^c^
	*Artemisia absinthium* L. leaf	I	AbL1	229.68 ± 0.16 ^c^	323.66 ± 0.02 ^d^	57.09 ± 0.01 ^e^	253.39 ± 0.01 ^f^
	*Artemisia absinthium* L. stem	I	AbS1	51.73 ± 0.11 ^f^	293.51 ± 0.01 ^e^	110.77 ± 0.03 ^d^	1314.38 ± 0.01 ^b^
			Sig.	***	***	***	***

Values are expressed as the mean of two replicates. Values with different letters in the same column indicate statistically significant (Sig.) differences (Tukey’s test, *p* < 0.05). *** *p* < 0.001; TPC—total phenolic content; GAE—gallic acid equivalents; DW—dry weight; DPPH—2,2-diphenyl-1-picrylhydrazyl; ABTS—2,2′-azino-bis (3-ethylbenzothiazoline-6-sulfonic acid).

**Table 2:** Pearson correlation coefficients for each pair of total phenolic content (TPC), DPPH (2,2′-diphenyl-1-picrylhydrazyl radical) and ABTS (2,2′-azinobis (3-ethylbenzothiazoline-6-sulfonatesulfonic acid)) in wormwood leaf and stem.

Correlation between		R	p	N
TPC ^total samples^	DPPH ^total samples^	0.959	0.0001 ***	18
TPC ^total samples^	ABTS ^total samples^	0.421	0.082 ^ns^	18
TPC ^leaf samples^	DPPH ^leaf samples^	0.967	0.0001 ***	9
TPC ^stem samples^	DPPH ^stem samples^	−0.949	0.0001 ***	9

R—Pearson correlation coefficient; p—is the probability of obtaining an F-ratio as large or larger than the one observed, assuming that the null hypothesis of no difference amongst group means is true; N—number of samples. Significance of effect: ns—not significant, *p* > 0.05; *** extremely significant *p* ≤ 0.001.

**Table 3:** Amounts of polyphenolic compounds in wormwood ethanolic aerial extracts (µg/mL).

Sample	Gallic Acid	Catechin	Vanillic Acid	Caffeic Acid	Epicatechin	p-Coumaric Acid	Ferulic Acid	Sinapic Acid	Rutin	Quercetin	Luteolin	Sig.
**AnL**	1.132 ± 0.001 ^a I^	16.603 ± 0.001 ^a E^	46.863 ± 0.002 ^b C^	nd	4.957 ± 0.002 ^d F^	51.267 ± 0.002 ^a B^	1.575 ± 0.002 ^a G^	285.694 ± 0.002 ^a A^	17.320 ± 0.000 ^a D^	1.653 ± 0.003 ^b G^	1.218 ± 0.002 ^a H^	***
**AnS**	0.086 ± 0.004 ^b H^	8.003 ± 0.003 ^b B^	6.502 ± 0.003 ^e C^	nd	1.914 ± 0.001 ^e D^	6.549 ± 0.001 ^b C^	0.669 ± 0.001 ^b F^	44.155 ± 0.001 ^b A^	0.519 ± 0.001 ^b G^	nd	0.703 ±0.001 ^c E^	***
**AbL2**	nd	1.275 ± 0.001 ^d C^	66.777 ± 0.002 ^a A^	nd	10.136 ± 0.002 ^c B^	1.375 ± 0.002 ^d C^	nd	nd	nd	nd	nd	***
**AbS2**	nd	nd	11.913 ± 0.002 ^d B^	nd	21.123 ± 0.001 ^a A^	0.248 ± 0.001 ^e D^	0.145 ± 0.002 ^e E^	0.267 ± 0.002 ^c D^	nd	nd	1.035 ± 0.002 ^b C^	***
**AbL1**	nd	1.262 ± 0.002 ^d D^	42.241 ± 0.001 ^c A^	nd	13.488 ± 0.001 ^b B^	2.565 ± 0.002 ^c C^	0.227 ± 0.002 ^d E^	nd	nd	nd	nd	***
**AbS1**	nd	2.438 ± 0.002 ^c B^	2.420 ± 0.002 ^f B^	nd	1.600 ± 0.002 ^e C^	0.359 ± 0.002 ^e E^	0.121 ± 0.002 ^e F^	nd	nd	8.492 ± 0.002 ^a A^	1.046 ± 0.002 ^b D^	***
**Sig.**	***	**	***		***	***	**	***	***	***	**	

Values are expressed as the mean of two replicates. Values with different letters in the same column indicate statistically significant (Sig.) differences (Tukey’s test, *p* < 0.05). ** *p* < 0.01; *** *p* < 0.001; ns *p* > 0.05, not significant. Different capital letters within rows indicate very significant differences between compounds (*p* < 0.01). nd—not detected.

**Table 4:** Assessment of the minimum inhibitory concentration and the minimum bactericidal concentration values of the wormwood ethanolic extracts from different growing years.

Sample	*Staphylococcus aureus* ATCC 25923		*Escherichia coli* ATCC 25922		*Listeria monocytogenes* ATCC 19114		*Salmonella enteritidis* ATCC 13076	
	MIC (mg/mL)	MBC (mg/mL)	MIC (mg/mL)	MBC (mg/mL)	MIC (mg/mL)	MBC (mg/mL)	MIC (mg/mL)	MBC (mg/mL)
**AnL**	2.00 ± 0.014 ^f^	5.00 ± 0.014 ^e^	5.00 ± 0.014 ^e^	12.00 ± 0.014 ^f^	5.00 ± 0.014 ^e^	5.00 ± 0.014 ^f^	5.00 ± 0.014 ^e^	12.00 ± 0.014 ^c^
**AnS**	54.00 ± 0.002 ^d^	114.00 ± 0.014 ^c^	54.00 ± 0.014 ^d^	114.00 ± 0.014 ^d^	114.00 ± 0.014 ^c^	114.00 ± 0.014 ^d^	54.00 ± 0.002 ^d^	241.00 ± 0.014 ^a^
**AbL2**	25.00 ± 0.002 ^e^	54.00 ± 0.014 ^d^	54.00 ± 0.014 ^d^	54.00 ± 0.002 ^e^	54.00 ± 0.002 ^d^	54.00 ± 0.002 ^e^	54.00 ± 0.002 ^d^	54.00 ± 0.002 ^b^
**AbS2**	114.00 ± 0.014 ^a^	114.00 ± 0.014 ^c^	240.00 ± 0.014 ^c^	240.00 ± 0.014 ^c^	114.00 ± 0.014 ^c^	240.00 ± 0.014 ^b^	240.00 ± 0.014 ^c^	-
**AbL1**	89.50 ± 0.028 ^b^	255.00 ± 0.014 ^b^	255.00 ± 0.014 ^b^	255.00 ± 0.014 ^b^	121.00 ± 0.014 ^b^	121.00 ± 0.014 ^c^	255.00 ± 0.014 ^b^	-
**AbS1**	85.00 ± 0.002 ^c^	375.00 ± 0.014 ^a^	375.00 ± 0.014 ^a^	375.00 ± 0.014 ^a^	178.00 ± 0.014 ^a^	375.00 ± 0.014 ^a^	375.00 ± 0.014 ^a^	-
**Gentamicin**	0.0005		0.00024		0.00152		0.00024	
**Sign.**	***	***	***	***	***	***	***	**

Values are expressed as the mean of two replicates. Values with different letters in the same column indicate statistically significant (Sig.) differences (Tukey’s test, *p* < 0.05). ** *p* < 0.01; *** *p* < 0.001; *p* 0.05, not significant; MIC—minimum inhibitory concentration; MBC—minimum bactericidal concentration.

**Table 5:** Inhibition zones induced by wormwood against bacterial strains (mm).

Sample	*Klebsiella* spp. ESBL1	*Klebsiella* spp. ESBL 2	*Klebsiella* spp. ESBL 3	*Klebsiella* spp. ESBL 4	*Klebsiella* spp. ESBL 5	*Klebsiella* spp. CRE 1	*Klebsiella* spp. CRE 2	*Klebsiella* spp. CRE 3	*Klebsiella* spp. CRE 4	*Klebsiella* spp. CRE 5	*E. coli* spp. ESBL 1	*E. coli* spp. ESBL 2	*E. coli* spp. ESBL 3	*E. coli* spp. ESBL 4	*E. coli* spp. ESBL 5
**AnL**	10.863 ± 0.308 ^a^	5.473 ± 0.756 ^a^	8.866 ± 2.0304 ^ab^	6.846 ± 0.170 ^b^	5.840 ± 0.215 ^bc^	12.756 ± 0.993 ^a^	11.120 ± 3.187 ^a^	7.210 ± 0.441 ^a^	7.210 ± 0.891 ^a^	6.876 ± 0.732 ^a^	5.117 ± 1.366 ^a^	8.610 ± 1.861 ^a^	4.753 ± 1.467 ^a^	7.946 ± 1.788 ^a^	8.130 ± 1.731 ^a^
**AnS**	2.523 ± 0.606 ^c^	1.920 ± 0.13 ^ef^	3.593 ± 0.472 ^cd^	5.106 ± 0.783 ^bc^	4.993 ± 0.248 ^c^	9.843 ± 0.945 ^b^	7.180 ± 0.669 ^b^	1.796 ± 0.248 ^ab^	3.286 ± 0.177 ^b^	5.020 ± 0.157 ^b^	4.473 ± 0.315 ^a^	4.100 ± 0.845 ^b^	5.67± 0.682 ^a^	3.656 ± 0.708 ^b^	4.583 ± 0.724 ^ab^
**AbL2**	1.083 ± 0.145 ^d^	3.026 ± 0.248 ^de^	2.360 ± 0.964 ^d^	2.330 ± 1.598 ^d^	1.926 ± 0.516 ^d^	1.756 ± 0.273 ^c^	0.593 ± 0.639 ^c^	5.340 ± 4.624 ^ab^	1.146 ± 1.986 ^b^	0.423 ± 0.733 ^c^	3.486 ± 3.080 ^a^	0.423 ± 0.733 ^c^	0.403 ± 0.698 ^b^	0.310 ± 0.536 ^c^	0.056 ± 0.098 ^b^
**AbS2**	4.493 ± 0.609 ^b^	1.410 ± 0.75 ^f^	5.610 ± 0.745 ^bcd^	3.950 ± 1.565 ^cd^	3.353 ± 0.606 ^cd^	0.313 ± 0.542 ^c^	0.206 ± 0.357 ^c^	2.016 ± 0.919 ^ab^	0.400 ± 0.560 ^b^	0.123 ± 0.213 ^c^	3.136 ± 2.738 ^a^	0.426 ± 0.739 ^c^	0.170 ± 0.294 ^b^	0.243 ± 0.421 ^c^	0.466 ± 0.808 ^b^
**AbL1**	1.583 ± 0.282 ^cd^	5.183 ± 0.620 ^ab^	6.793 ± 1.116 ^bc^	3.803 ± 0.329 ^cd^	10.110 ± 1.68 ^a^	0.313 ± 0.542 ^c^	0.136 ± 0.236 ^c^	1.206 ± 0.780 ^b^	1.003 ± 0.976 ^b^	0.053 ± 0.092 ^c^	2.626 ± 2.277 ^a^	0.336 ± 0.583 ^c^	0.430 ± 0.744 ^b^	0.683 ± 1.183 ^c^	3.430 ± 5.94 ^ab^
**AbS1**	2.180 ± 0.448 ^cd^	3.833 ± 0.225 ^bc^	11.246 ± 1.71 ^a^	11.000 ± 0.245 ^a^	8.436 ± 1.699 ^ab^	0.210 ± 0.363 ^c^	0.126 ± 0.219 ^c^	0.390 ± 0.675 ^b^	1.476 ± 0.958 ^b^	0.096 ± 0.167 ^c^	4.723 ± 0.895 ^a^	1.270 ± 0.208 ^c^	0.053 ± 0.092 ^b^	0.466 ± 0.808 ^c^	0.170 ± 0.294 ^b^
**Sig.**	ns	ns	ns	**	ns	ns	***	***	*	**	ns	*	*	ns	*

Values are expressed as the mean of two replicates. Values with different letters in the same column indicate statistically significant (Sig.) differences (Tukey’s test, *p* < 0.05). * *p* < 0.05; ** *p* < 0.01; *** *p* < 0.001; *p* > 0.05, ns: not significant. ESBL, extended-spectrum β-lactamases; CRE, carbapenem-resistant enterobacteriaceae.

## Data Availability

The data presented in this study are openly available in FigShare at https://figshare.com/articles/figure/Antibacterial_and_Phytochemical_screening_of_Artemisia_species/21937217 (accessed on 22 January 2023).

## Supplementary Materials

The following supporting information can be downloaded at: https://www.mdpi.com/article/10.3390/antiox12030596/s1.
