# Supplementary material for: Antibacterial and Phytochemical Screening of Artemisia Species
Source: Antioxidants (Basel). 2023 Feb 27;12(3):596. doi: 10.3390/antiox12030596 (PMC10045255; doi:10.3390/antiox12030596)
Supplement: Supplementary file 1 [file antioxidants-12-00596-s001.zip › antioxidants-2140034-supplementary.pdf]

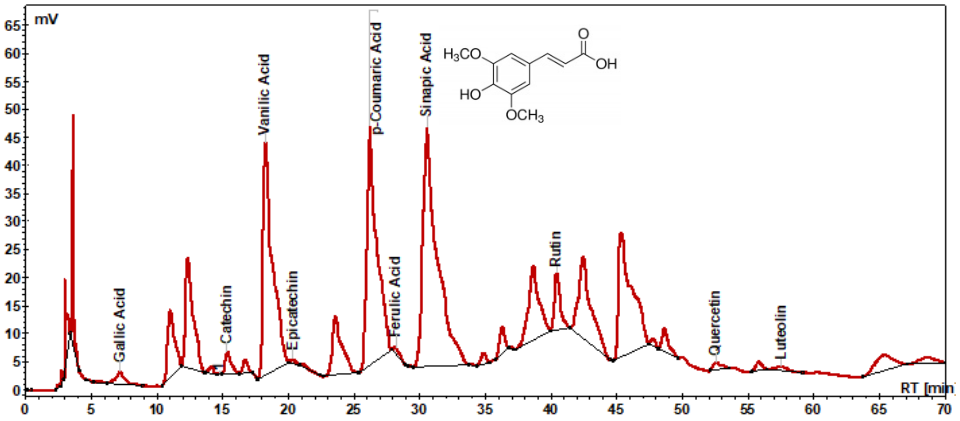

a. AnL

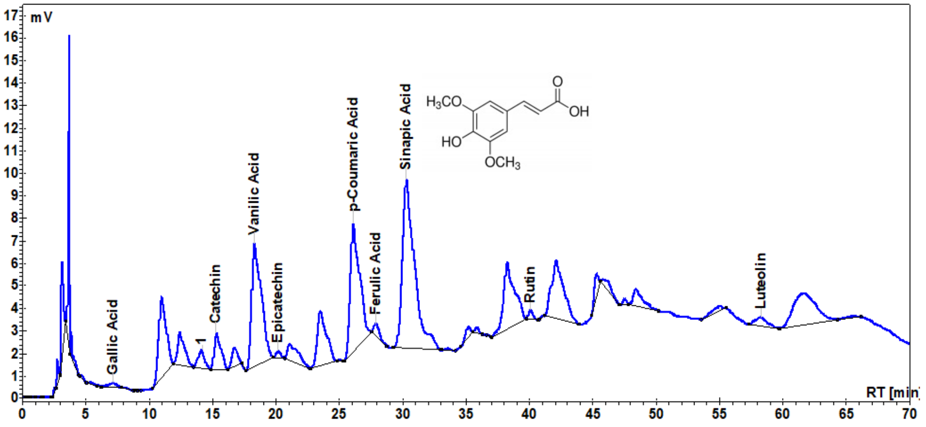

b. AnS

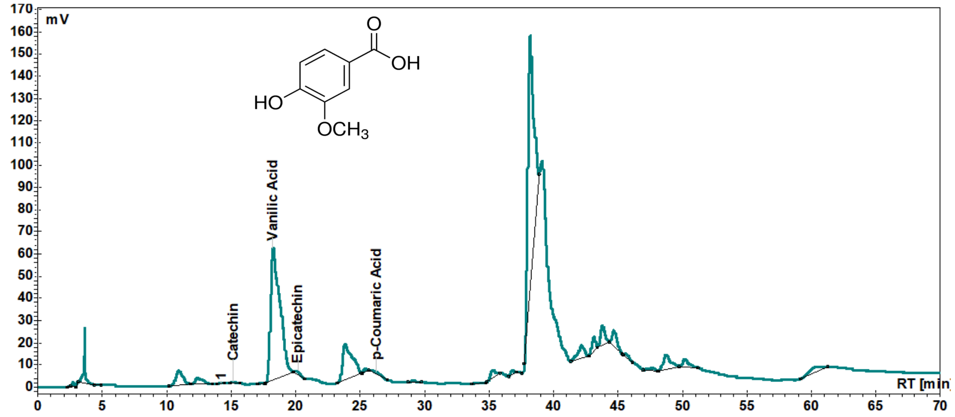

c. AbL2

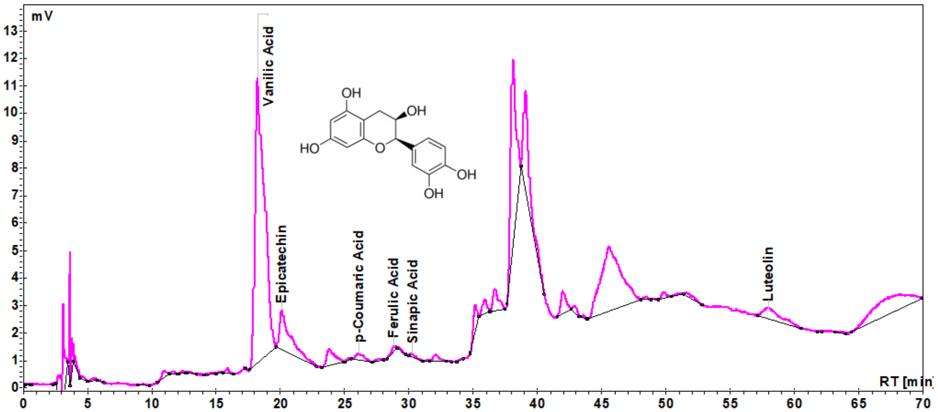

d. AbS2

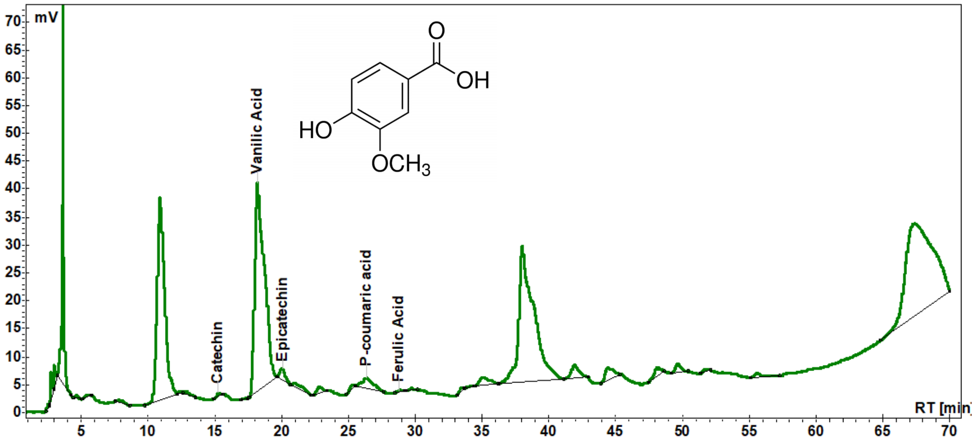

e. AbL1

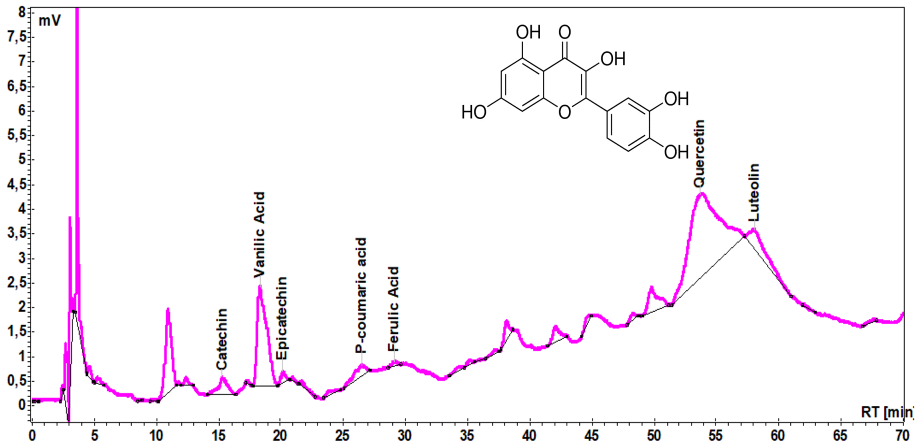

f. AbS1

Figure S1. HPLC chromatograms of the polyphenols content in wormwood ethanolic aerial parts. (AnL - *Artemisia annua* L. leaf, AnS - *Artemisia annua* L. stem, AbL2 - *Artemisia absinthium* L. leaf, AbS2 - *Artemisia absinthium* L. stem, AbL1 - *Artemisia absinthium* L. leaf, AbS1 - *Artemisia absinthium* L. stem)
